# Supplementary material for: New craniodental remains of Wakaleo alcootaensis (Diprotodontia: Thylacoleonidae) a carnivorous marsupial from the late Miocene Alcoota Local Fauna of the Northern Territory, Australia
Source: PeerJ. 2015 Nov 12;3:e1408. doi: 10.7717/peerj.1408 (PMC4647553; doi:10.7717/peerj.1408)
Supplement: Supplemental Information 1 [file peerj-03-1408-s001.docx]

#NEXUS

BEGIN TAXA;

DIMENSIONS NTAX=10;

TAXLABELS

Pseudocheirus

Nimiokoala

Namilamideta

roskellyae

oldfieldi

vanderleueri

alcootaensis

hilli

crassidentatus

carnifex

;

ENDBLOCK;

BEGIN CHARACTERS;

DIMENSIONS NCHAR=34;

FORMAT DATATYPE=STANDARD MISSING=? GAP=- SYMBOLS="01234";

;

MATRIX

Pseudocheirus 2000000000 0000010?00 0000000000 0000

Nimiokoala 0001000000 0000000?00 0110000000 0?00

Namilamideta 0101100000 0010011000 0110000000 0000

roskellyae ????2????? ??10111000 0001010010 ??00

oldfieldi 1211211110 0111111101 1??1121110 01?1

vanderleueri 1211211110 0121111101 11{01}1122110 0011

alcootaensis 03??21?110 1121111101 11111?211? 1110

hilli 2?10?????? ???220001? ???1?????? 0???

crassidentatus 23002222?1 1132200010 1001020??1 00??

carnifex 23002222?1 1132200010 100102???1 101?

;

ENDBLOCK;

BEGIN ASSUMPTIONS;

OPTIONS DEFTYPE=UNORD POLYTCOUNT=MINSTEPS;

TYPESET * default = ORD: 2 6 - 8 13 - 14 26 - 27;

ENDBLOCK;
